# Perspectives on translational molecular imaging and therapy: an overview of key questions to be addressed

**DOI:** 10.1186/s13550-022-00903-0

**Published:** 2022-06-02

**Authors:** Fijs W. B. van Leeuwen, Margret Schottelius, Felix M. Mottaghy, Fabien Hyafil, Mark Lubberink, Gabriela Kramer-Marek, Wim J. G. Oyen

**Affiliations:** 1 Interventional Molecular Imaging Laboratory, Department of Radiology, Leiden University Medical Center, Leiden, The Netherlands; 2 Unit of Translational Radiopharmaceutical Sciences, Departments of Nuclear Medicine and of Oncology, Centre Hospitalier Universitaire Vaudois (CHUV) and University of Lausanne (UNIL), Lausanne, Switzerland; 3 Department of Nuclear Medicine, University Hospital, RWTH Aachen University, Aachen, Germany; 4 Department of Radiology and Nuclear Medicine, Maastricht University Medical Center (MUMC+), Maastricht, The Netherlands; 5 Department of Nuclear Medicine, DMU IMAGINA, APHP, Hôpital Européen Georges-Pompidou, University of Paris, Paris, France; 6 PARCC, INSERM 690, Université de Paris, Paris, France; 7 Radiology and Molecular Imaging, Department of Surgical Sciences, Uppsala University, Uppsala, Sweden; 8 Department of Medical Physics, Uppsala University Hospital, Uppsala, Sweden; 9 Division of Radiotherapy and Imaging, The Institute of Cancer Research, London, UK; 10 Humanitas Clinical and Research Center, Humanitas University, Milan, Italy; 11 Department of Radiology and Nuclear Medicine, Rijnstate Hospital Arnhem, Arnhem, The Netherlands; 12 Department of Radiology and Nuclear Medicine, Radboud University Medical Centre, Nijmegen, The Netherlands

With the increasing insight into the complexity of diseases along with the growing number of options for treatments, molecular imaging is needed to improve the in vivo phenotyping of diseases and to support the shift toward personalized medicine, i.e., tailored therapeutic strategies.

The fields of nuclear medicine and molecular imaging include a wealth of different technologies and methodologies, that go beyond PET and SPECT imaging devices and radiopharmaceuticals. The term “technologies” should thus be considered from a multidisciplinary point of view and in very broad terms that entail (1) (radio)chemistry, (2) molecular biology and genetics tools, (3) engineering of modalities (e.g., detectors, scanners), (4) software engineering (e.g., pharmacokinetics, image processing or artificial intelligence), (5) rational design of new (patient-friendly) treatment paradigms, and follow-up or surveillance techniques. It is a unique feature of molecular imaging that these innovations can be implemented in a wide variety of clinical disciplines, among others oncology, cardiology and neurology. Furthermore, such technologies may be applied for diagnostic as well as therapeutic applications, supporting the development of theranostics [1], a concept that arose 90 years ago when radio-iodine was first used [2] and now extends to vectorized internal radiotherapy with, for example, alpha-emitting targeted agents [3]. The translational process for a technology (or combinations thereof) requires a succession of hurdles to be overcome. The variation between technologies and their applications renders efforts to design “one-size-fits-all” recipe for translational success quite challenging, if not impossible. Nevertheless, looking back on past translational success stories, we can identify some central elements that make translational science successful and sustainable.

In this editorial, based on cornerstones for going the entire way from “bench to bedside” (and back), we address some key questions which we consider valuable or even critical for translational research.

## The primary questions in translational science

### On behalf of whom do we translate new technologies and what challenges need to be solved?

This is a simple question, to which different answers may be appropriate. Without a doubt, the patient is the primary stakeholder in translational medicine, followed by medical teams treating and looking after their patients. Subsequently, there is a wide variety of parties involved, such as medical societies, hospital boards, academic institutions, industry, healthcare insurance companies, governments and society as a whole. Having many stakeholders can be considered beneficial when pursuing new technologies as it provides input from very different angles. Nevertheless, having multiple stakeholders also comes with challenges. These will become apparent when you approach different stakeholders with the question: what type of technology do you need? Most likely, patients will provide the most straightforward answers: “I want to get the best care possible, and I want to be cured of my disease at the expense of as few side effects as possible.” But beyond that, there are likely a lot of variations in the answers provided, and the answers may become quite complex and detailed. For example, from the perspective of technology developers, there is a real risk that technical feasibility starts to drive the generation of solutions for problems that may not necessarily be the problems faced by the primary stakeholders. Ultimately, this can mean the scientific focus shifts from the initially defined urgent clinical needs to problems faced by technology developers. Evidently, these aspects need to be balanced but given that the golden rule is “that you cannot solve a problem unless you understand it,” it is of paramount importance to maintain focus on the patient needs.

### Along which routes can we translate?

Many will say that translational medicine is about moving scientific innovations “from bench to bedside” or from “molecule to man” [4], and there are many more variations on this terminology. While very popular, such statements merely cover the technical aspects of translating technologies. As such, the statements may even harm the cause by suggesting that knowledge translation only occurs in a one-way direction. In fact, the success of most translational research appears to be the result of a back-and-forth interaction between team members with different, but complementary backgrounds. This interaction is driven by a continuous feedback loop between preclinical innovations and new clinical questions that arise during the use of established and new technologies. Furthermore, the radius of such a feedback loop may extend over time, including additional disciplines. The increasing impact of radiobiology research in the context of nuclear theranostics is one key example [5]. To achieve long-term impact, we need to learn, understand and appreciate needs, expertise and views of the other “key players” and disciplines. Basically, we continuously need to educate ourselves as professionals and should look beyond artificial barriers set by mono-disciplinarity, while continuously reaching out to the public and patient organizations.

In (radio)pharmaceutical development, compound design and synthesis need to be followed by in situ studies (stability), in vitro studies (affinity, cellular uptake, early toxicity), ex vivo studies (specificity, signal intensity), and in vivo studies in models of disease (specificity, pharmacokinetics, dosing and timing). Alternatively, efforts may focus on repurposing existing pharmaceuticals [6]. Despite the widespread focus on (radio)pharmaceuticals, medical device development (hardware and software) are equally important. These, however, have different legal requirements [7]. Examples are the development of radiosynthesis modules, detector crystals, (whole body) PET, etc. In such developments, laboratory studies using, for example, phantoms or datasets are preferably extended with studies in animals that are size-matched to humans. Unique for software is that the so-called digital-twinning can be used to evaluate novel image processing algorithms in a clinical setting [7, 8]. In general, before moving to clinical trials, technologies need to be compliant to good manufacturing practice (GMP) or medical device regulations (MDR) [7, 9] and safety needs to be established [10].

In radiopharmaceutical development, thanks to the pico- to nano-molar sensitivities for detecting most tracers, i.e., at concentrations several times lower than the ones associated with pharmacological effects, novel diagnostic compounds can be used within the framework of a micro-dosing regimen (≤ 100 µg/patient) [11]. This limits the required toxicity studies and thus helps reduce cost. Here, it must be noted that recent developments in therapeutic and immune imaging applications indicate that in some cases radiopharmaceuticals are best applied close to a therapeutic dose. In dependent of the dosing, (long term) validation of technologies in (randomized) clinical trials is the final step toward regulatory approval (European Medicine Agency (EMA) or medical CE marking) before application in daily clinical practice. These legislation-based “step by step” aspects provide a framework for translational routes.

### When can translation be considered a success?

Although a highly relevant question, it may again be answered differently, largely depending on whom we ask. If we argue that patients and medical professionals should benefit from a technology, successful translation means that a technology surpasses the first-in-human stage and shows benefit to the patient and/or healthcare professional in prospective clinical trials, ultimately making it into daily routine clinical care. This significantly reduces the number of “real” translational success stories and makes the effort highly complex and expensive. Since successful technologies need to mature from lead compounds/prototypes to approved drugs/medical devices that are safe to use in patients, many regulations and requirements need to be met. Making translational medicine a very costly and time-consuming journey or even ordeal. Especially considering that, late clinical failure comes with a loss of substantial investment. To demonstrate the potential and value of an investment, a technology should have a business case that documents its market potential. Products, however, only have market value when there is high-level evidence demonstrating their benefit for the intended patients and/or professionals (ideally going beyond existing care) and if a widespread application of the new technology can be anticipated.

### How critical is intellectual property (IP)?

IP provides security for companies, can drive up-front investments and can help establish enough financial backing to fund costly phase III trials. An example here is [^177^Lu]PSMA–617 [12, 13]. It is important to note that, while such investments may help overcome hurdles and may drive the marketing potential, they may also come with financial interests. Basically, private entities “spend money to make money.” Perhaps trivial to mention, but financial commitment by no means provides a base for the realization of a marketing authorization. In contrast, there are highly successful, IP-free, academic success stories where translation has occurred in an unconventional way, i.e., by nonprofit efforts that generally revolve around in-house production in an academic setting. Recent examples are the prostate-specific membrane antigen (PSMA)-targeted tracers ^68^ Ga-PSMA-11 [14] and ^99m^Tc-PSMA I&S [15]. These divergent routes (IP-covered or IP-free) may also merge at some point, since commercial entities can also obtain marketing authorization for products developed in an academic setting. Examples are FDA/EMA-approved pharmaceuticals such as ^18^F-FDG [16, 17] and ^177^Lu-oxodotreotide (Lutathera®) [18]. An example of a medical device is found in the DROP-IN gamma probe [19], which moved from investigator-initiated research to a CE-marked product. Combined, this suggests that IP on new technologies certainly helps to establish financial backing but is not critical.

### Which type of clinical trials is needed to define success?

Going beyond first-in-human applications, which are limited to providing proof-of-concept data, automatically means that higher patient numbers and high-end output data are needed. In nuclear medicine, this intrinsically means entering the discussion on the value of retrospective studies in which a new technology has been implemented under compassionate use procedures. This approach is common in several European countries, especially when using radiotracers within micro-dosing regimens. While no one can argue against the positive effect that such work has had on evaluating the potential of novel radiopharmaceuticals in humans at an early stage, acceptance of a technology in clinical routine more often requires (randomized) prospective validation studies. This means that public efforts often rely on the financial backing of private partnerships to reach implementation in routine care, as has been shown by the examples of Lutathera® and PSMA-11 in the previous paragraph. At the same time, one may argue that it is undesirable to have industry influence strategical decision-making in academia. In this respect, we should remain cautious not to let industrially backed technologies limit academic innovation and translation.

While most trials focus on patient benefit, we also postulate that benefits for the professionals represent an interesting value on their own. One prime example can be found in image-guided surgery. Here, one may argue that an imaging technology that facilitates and improves surgical performance and precision can be considered successful.

### Which ethical aspects need to be considered?

In science and healthcare, practical requirements can unfortunately also become mixed with other considerations such as economic or political benefits. In extreme cases, such distractions can become so dominant that patients are no longer the primary beneficiaries. But even having the patient in mind does not automatically mean that an experimental technology will prove its value and move forward in the translational pipeline. This raises the question: can we predict (late) clinical failure? This is a tough question to answer, but from an ethical point of view a question that needs to be considered, especially given the widespread drive to realize first-in-human translation. Is it ethically justifiable to test novel technologies, for which potential “success” may not be immediately anticipated? Or is it justifiable to pursue new strategies when there are good alternatives? With regard to the last, the MDR already state that experimental technologies should not be used when CE-marked alternatives are available for the same indication [20].

These ethical questions start to become relevant very early in the development pipeline. For example, when pursuing animal experiments in the preclinical test phase, most new imaging agents are extensively tested in animal models. Obviously, such experiments need to be minimized and should only be conducted with technologies that hold sufficient potential. When preclinical studies have a positive outcome, they need to be followed by toxicity/safety studies [21, 22], often conducted by specialist companies. In extension of the toxicity/safety, we should ask ourselves what we define as a valid performance threshold for first-in-man studies? While addressing this question, we need to leave enough room for high potential innovations, but at the same time avoid exploitation of patients for a “first-in-man” paper.

One may argue that ultimately benefit is only created when all the above questions on translational goals and translational successes are satisfactorily answered. Failing to do so may rightfully challenge the pursuit of certain technologies. With the increasing regulatory hurdles, the role of financing has also become an increasingly dominant factor in the failing of translational efforts. Non-profit-oriented public efforts with limited research budgets compared to private efforts are, however, instrumental for the future of translational medicine. This means academic researchers need to establish innovative approaches that strike a reasonable balance between innovation, ethics, legislation, and financial burden.

## Conclusion

In translational molecular imaging as much as in other domains, there are many roads leading to Rome. Independently of the translational route selected, some essential challenges need to be met for a technology to be integrated into clinical care. This demands a multidisciplinary approach. In the context of clinical translation of novel technologies in nuclear medicine and molecular imaging, the most critical aspects are often the financial aspects and the balance struck between (technological) innovations and potential clinical benefits. We, as the editorial team, with our diverse preclinical and clinical backgrounds, see tremendous value and potential for innovation in molecular imaging, theranostics and image-guided therapy. We are passionate and excited about this vibrant field of research that keeps bringing forth new technologies that can transform into breakthrough discoveries. Our aim is that *EJNMMI Research* becomes a home for high-quality translational research in nuclear medicine and molecular imaging. In doing so, we intent to stimulate multidisciplinary interactions that drive the emergence of new ideas and concepts.

## Data Availability

Not applicable.
